# Whey protein improves glycemia during an oral glucose tolerance test compared to vigorous-intensity aerobic exercise in young adult men

**DOI:** 10.1186/s13102-022-00540-z

**Published:** 2022-07-30

**Authors:** Ryan A. Gordon, Emily L. Zumbro, Todd J. Castleberry, Matthew L. Sokoloski, Matthew F. Brisebois, Christopher J. Irvine, Anthony A. Duplanty, Vic Ben-Ezra

**Affiliations:** 1 Department of Biology, Drury University, Springfield, MO USA; 2 Department of Medicine, University of Alabama at Birmingham, Birmingham, AL USA; 3 UAB Center for Exercise Medicine, University of Alabama at Birmingham, Birmingham, AL USA; 4 Department of Kinesiology, Louisiana Tech University, Ruston, LA USA; 5 School of Health Promotion and Kinesiology, Texas Woman’s University, Denton, TX USA; 6 Department of Human Performance and Health, University of South Carolina Upstate, Spartanburg, SC USA; 7 Department of Health and Human Performance, Rocky Mountain College, Billings, MT USA

**Keywords:** Whey, Exercise, Glucose, Insulin, Incretins, Glycemia

## Abstract

**Background:**

Both aerobic exercise and whey protein can improve glucose regulation. The purpose of this study was to investigate how a single bout of vigorous-intensity aerobic exercise and whey protein, independently, as well as when combined, influence glycemia during an oral glucose tolerance test in sedentary, young men.

**Methods:**

Healthy males (*n* = 11) completed four randomized trials: no exercise/no whey protein (R); exercise (EX; walking at 70% VO_2max_ for 60 min); 50 g of whey protein (W); and exercise combined with 50 g of whey protein (EXW). Each trial included a 75 g oral glucose tolerance test (OGTT) that was completed after an overnight fast. Blood samples were collected over a two-hour period during the OGTT. For EX and EXW, the exercise was performed the evening before the OGTT and the 50 g of whey protein was dissolved in 250 mL of water and was consumed as a preload 30 min prior to the OGTT. For R and EX, participants consumed 250 mL of water prior to the OGTT. Plasma samples were analyzed for glucose, insulin, C-peptide, glucagon, gastric inhibitory peptide (GIP) and glucagon like peptide 1 (GLP-1), and postprandial incremental area under the curve (iAUC) was calculated for each.

**Results:**

Glucose iAUC was reduced during W (− 32.9 ± 22.3 mmol/L) compared to R (122.7 ± 29.8 mmol/L; *p* < 0.01) and EX (154.3 ± 29.2 mmol/L; *p* < 0.01). Similarly, glucose iAUC was reduced for EXW (17.4 ± 28.9 mmol/L) compared to R and EX (*p* < 0.01 for both). There were no differences in iAUC for insulin, C-peptide, GIP, GLP-1, and glucagon between the four trials. Insulin, C-peptide, glucagon, GIP, and GLP-1 were elevated during the whey protein preload period for W and EXW compared to EX and R (*p* < 0.01). There were no differences for insulin, C-peptide, glucagon, GIP, or GLP-1 between trials for the remaining duration of the OGTT.

**Conclusions:**

Glucose responses during an oral glucose tolerance test were improved for W compared to EX. There were no additional improvements in glucose responses when vigorous-intensity aerobic exercise was combined with whey protein (EXW).

## Background

Aerobic exercise, along with changes in diet, are primary strategies for improving blood glucose management [1]. Acute aerobic exercise improves glycemia through intracellular signaling within skeletal muscle [2]. This results in the activation of 5’-adenosine monophosphate-activated protein kinase (AMPK) and calcium/calmodulin-dependent protein kinase (CaMK) II, each of which influences GLUT4 translocation and expression, promoting an increase in cellular glucose uptake [3–5]. This increase in glucose uptake can persist for several hours in response to a single bout of aerobic exercise, and the change in glucose uptake post-exercise is dependent on the intensity, duration, and type of exercise performed [6, 7]. Following exercise, skeletal muscle also exhibits enhanced insulin sensitivity, and this has been shown to persist for 24 to 48 h post-exercise [3, 6, 8–10]. This enhanced insulin sensitivity contributes to improved glycemia while also reducing serum insulin values for 12 to 24 h following exercise [5, 11, 12]. The intensity and duration of endurance exercise influences the magnitude of its effects on blood glucose and insulin sensitivity post-exercise. Many studies have attempted to determine the optimal intensity and duration of endurance exercise to maximize its effects on managing blood glucose [11, 13, 14]. Many of these studies report positive effects occur when exercise is performed for 45 to 60 min at moderate to vigorous intensities (≥ 50% of VO_2max_).

Similar to aerobic exercise, nutritional strategies (e.g., whey protein) are also effective for improving blood glucose. Whey protein, when consumed prior to, or with a meal, improves postprandial glycemia [15–20]. These improvements in postprandial glycemia are partially due to whey protein’s ability to enhance insulin secretion [21–23]. Whey protein also stimulates the secretion of incretin hormones, glucagon-like peptide-1 (GLP-1) and gastric inhibitory peptide (GIP), which have been demonstrated to potentiate insulin secretion [21, 24–26]. Thus, whey protein’s ability to both directly and indirectly increase insulin secretion contributes to improved postprandial glycemia.

The timing of whey protein consumption is important when examining its effects on postprandial glycemia. Consuming whey protein (10 or 20 g) 30 min prior to a meal (i.e., preload) has been demonstrated to slow gastric emptying and increase GLP-1 secretion [18]. In a similar study, Gunnerud et al. (2012) found that 9 g of whey protein consumed as a preload immediately prior to a mixed meal reduced postprandial plasma glucose responses (iAUC) in the first 60 minutes [17]. The authors attributed this effect to increased insulin secretion as a result of the whey protein consumption. In addition to timing, the dose or amount of whey protein consumed appears to influence the magnitude of its effects on blood glucose [21]. Several studies have demonstrated positive effects on insulin and glucose responses with higher doses (20 – 55 g) of whey protein [19, 23, 27, 28]. Our lab has previously examined how differences in the dose of whey protein can influence glycemia. We found greater improvements in glycemic responses during a 75 g oral glucose tolerance test (OGTT) when 30 g of whey protein, compared to 20 g, was consumed 30 min prior to the OGTT [29]. Thus, whey protein’s positive effects on glycemia may be dose dependent, and its effects on postprandial glycemia may be maximized when the whey protein is consumed as a preload (e.g., 30 min prior to a meal or beverage with a high glycemic load). Several studies using similar designs reported improvements in measured variables using 50 g of whey protein [27, 28]. Based upon our observations that whey protein’s effects may be dose-dependent, we hypothesized a large dose of whey protein (i.e., 50 g) would result in greater improvements in postprandial glycemia compared to lower doses.

Both acute aerobic exercise and whey protein consumption improve blood glucose. Each may be effective individually, but their effects when combined are not fully understood within human subjects. Thus, the aim of this study was to investigate the individual and combined effects of a single bout of aerobic exercise and a whey protein preload on glycemic responses following an oral glucose tolerance test. When considering whey protein’s ability to increase insulin secretion and exercise’s effects on insulin sensitivity, we hypothesized that acute vigorous-intensity aerobic exercise (70% of VO_2max_; performed the previous day, 12 to 14 h before the whey protein consumption) in combination with a 50 g preload of whey protein prior to an oral glucose tolerance test would result in greater improvements in postprandial blood glucose responses when compared to whey protein or acute aerobic exercise alone.

## Methods

### Study population

Twelve apparently healthy, sedentary males aged 18 to 44 years were recruited for this study. Participants were excluded from data collection if they were prescribed medications that would influence blood glucose concentration (sulfonylurea or metformin), blood pressure medication (thiazide diuretics, angiotensin receptor blockers, angiotensin-converting enzyme inhibitors), or answered “Yes” to any question on the Physical-Activity Readiness Questionnaire (PAR-Q +) (2016) assessment. Participation in this study also required that participants perform less than 3 days per week of exercise, or less than 150 min of moderate-intensity cardiorespiratory exercise for the previous three months to be classified as sedentary according to American College of Sports Medicine criteria [30].

#### Participant screening

Prior to data collection, this study was approved by the Institutional Review Board of Texas Woman’s University (Protocol #: 19,806). In addition, prior to participation in this study, all participants were informed of the study purpose and procedures and gave their written informed consent. This study and its procedures were performed in accordance with the Declaration of Helsinki. Following explanation of the study and informed consent, a fasted (10–12 h) blood sample was collected from an antecubital vein and analyzed for plasma glucose concentration and hemoglobin A1C. Participants with a fasted blood glucose value ≥ 100 mg/dL were excluded from the study. Throughout the duration of this study, participants were required to keep three-day diet records detailing food and drink consumed prior to each trial. Though a total of twelve participants were recruited for the study, one participant dropped out of the study due to noncompliance, resulting in eleven participants completing the study. Anthropometrics and characteristics for participants are shown in Table 1 and dietary records reflecting caloric and carbohydrate intake 24-h prior to each trial are presented in Table 2.

**Table 1:** Participant descriptive characteristics

	Participants (*n* = 11)
Age (y)	24.3 ± 1.6
Height (cm)	179.3 ± 1.6
Weight (kg)	84.3 ± 6.0
BMI (kg/m^2^)	26.0 ± 1.7
HbA1c (%)	5.2 ± 0.1
Fasting plasma glucose (mmol/L)	4.73 ± 0.02
VO_2max_ (ml/kg/min)	38.3 ± 1.8
VO_2max_ (L/min)	3.1 ± 0.1
Average exercise HR (EX) (bpm)	160 ±13
Average exercise HR (EXW) (bpm)	160 ± 16
Average exercise VO_2_ (EX) (L/min)	2.2 ± 0.3
Average exercise VO_2_ (EXW) (L/min)	2.2 ± 0.5

Data are presented as mean ± SE

**Table 2:** 24-Hour dietary records prior to each trial

	R	EX	W	EXW
Caloric intake (kcal)	1827.9 ± 164.9	1956.2 ± 178.7	2178.1 ± 230.0	2211.7 ± 192.8
Carbohydrate (g)	100.1 ± 11.7	108.7 ± 10.5	103.0 ± 11.1	111.5 ± 12.3

Data are presented as mean ± SE

#### Anthropometrics

Height was measured using a stadiometer (Perspective Enterprises; Kalamazoo, MI, USA) and weight was measured to the nearest 0.1 kg using a digital scale (Tanita Corp.; Arlington Heights, IL, USA). From these measurements, body mass index (BMI) was calculated (kg/m^2^). Body composition was analyzed using a dual energy X-ray absorptiometry (DEXA) scan (General Electric Lunar DXA-Prodigy, Madison, WI, USA).

#### Maximal aerobic capacity test

Participants performed a graded exercise test to determine their cardiorespiratory fitness. This test was performed using the Bruce Protocol on a Quinton ST65 motor driven treadmill (Quinton®-Q Stress, Ventura, CA, USA) until exhaustion [31]. During this test, heart rate and rhythm were continuously monitored using a Quinton Q Stress 12-lead electrocardiograph (Welch Allyn™, Skaneateles Falls, NY, USA). Thirty-second averages of respiratory gas exchange were continuously collected through indirect calorimetry (ParvoMedics, Sandy, UT, USA). Attainment of maximal oxygen consumption (VO_2max_) was determined by a plateau in VO_2_ (≤ 150 ml/min) with an increase in workload, or a combination of achieving an RER greater than 1.1 and a maximal heart rate within 10 bpm of age-predicted heart rate max (220-age) [30].

#### Study design

**Fig. 1:**
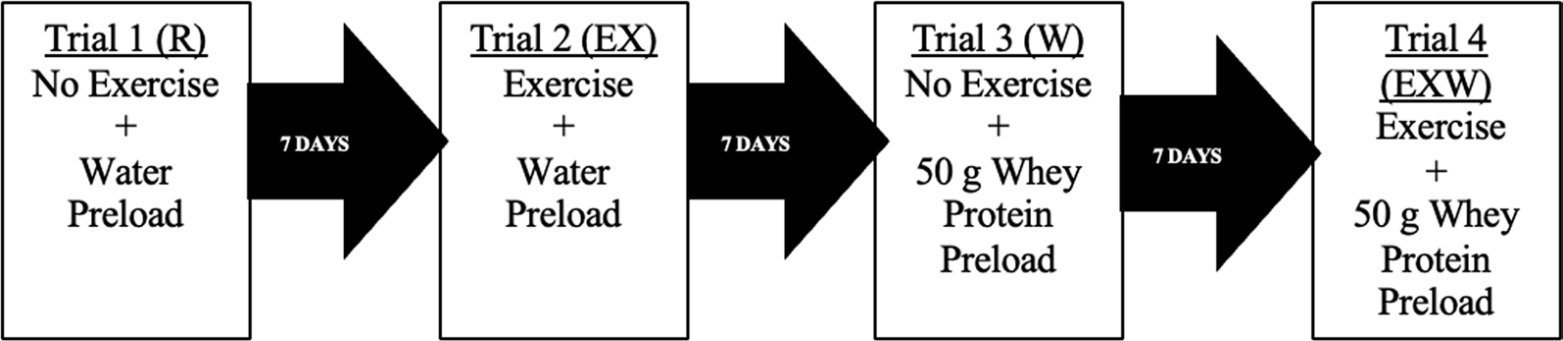
Representation of the randomly sequenced trials performed by participants

Participants in this study completed four trials, and each participant performed the sequence of trials in a randomized order (see Fig. 1 for example): Trial 1; no exercise and no whey protein prior to an OGTT (R). Trial 2; 60 min of exercise at 70% of VO_2max_ and no whey protein prior to an OGTT (EX). Trial 3; no exercise and a 50 g whey protein preload consumed prior to an OGTT (W). Trial 4; 60 min of exercise at 70% of VO_2max_ and a 50 g whey protein preload consumed prior to an OGTT (EXW). For trials in which exercise was performed, EX and EXW, the exercise session was performed 12–14 h before the start of the OGTT for that trial (i.e., the evening before). Trials for each participant were separated by a minimum of seven days, and participants were instructed to refrain from exercise or intense physical activity between each trial. Participants were also instructed to consume a meal similar in composition to their three-day diet records the evening prior to each OGTT.

#### Whey protein protocols

Whey protein isolate (50 g; Isopure® Unflavored WPI, Nature’s Best™, Hauppauge, NY, USA) was consumed as a preload 30 min prior to a 75 g OGTT for both W and EXW trials. The 50 g of whey protein was mixed with 250 ml of water for both W and EXW trials. For R and EX trials, participants were given 250 ml of water only as a control preload prior to their respective OGTTs.

#### Exercise or rest protocols

Exercise sessions were performed the evening prior to EX and EXW OGTTs (12–14 h before the OGTT). These sessions consisted of walking for 60 min at a speed and grade that achieved 70% of the participant’s VO_2max_.VO_2_ was measured for 60 s at three separate time intervals (5 min, 30 min, and 60 min) throughout the exercise duration to ensure the intensity was appropriate, with adjustments made to the speed or grade if necessary to maintain the target VO_2_. Heart rate was continuously monitored and recorded throughout the exercise session using a Polar H9 heart rate sensor (Polar Electro Inc., Bethpage, NY, USA).

#### Oral glucose tolerance test (OGTT) procedures

**Fig. 2:**
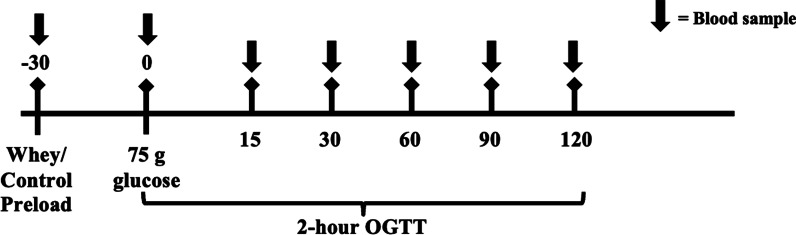
Timeline of preload beverage consumption and blood sample collection throughout the OGTT for each trial

Participants arrived fasted (10–12 h) to the Exercise Biochemistry Lab at Texas Woman’s University between 0600 and 0800 for all OGTTs. For each OGTT, a catheter was placed in an antecubital vein by a trained phlebotomist. A blood sample (5 mL) was collected and analyzed for fasted glucose to verify the participant was in the fasted state. Participants then consumed either the 50 g of whey protein preload (W and EXW) or water as a control beverage (R and EX). Participants were required to consume the whey or control beverage within five minutes. Thirty minutes after consuming the preload or water (i.e., control), a blood sample was collected. Immediately following this blood sample, participants consumed a commercial 75 g glucose tolerance test beverage (Trutol® Dextrose, ThermoFisher Scientific™ Inc., Waltham, MA, USA). Blood samples were then collected 15, 30, 60, 90, and 120 min following the consumption of the glucose tolerance test beverage (Fig. 2). A sterile saline drip was used to flush the catheter following each blood sample, with a drip rate of 1 drop per 4 s. Participants remained seated in a thermoneutral environment and were able to read or watch television throughout the duration of the OGTT.

#### Biochemical analysis

Blood samples were collected into 6 mL EDTA tubes containing a concentration of 1.25 mg Pefabloc® of blood (Roche Diagnostics, Mannheim, Germany) and 5 μL Protease Inhibitor (EMD Millipore™ Corporation, Billerica, MA, USA) per mL of blood. After collection, blood samples were immediately centrifuged at 3000 rpm for 10 min at 10 ℃. Plasma glucose was analyzed using a YSI 2900D glucose analyzer (Yellow Spring Inc, Yellow Springs, OH, USA). Plasma hormone concentrations of C-peptide, GIP, GLP-1, insulin, and glucagon were analyzed using a Luminex™ Human Metabolic Hormone multiplex assay (HMHEMAG-34, EMD Millipore™, Billerica, MA, USA).

#### Statistical analysis

Postprandial incremental area under the curve (iAUC) was calculated from timepoints 0 to 120 min (OGTT) using the trapezoidal method for glucose, insulin, C-peptide, GIP, GLP-1, and glucagon. Repeated measures analysis of variance (ANOVA) was used to examine differences in iAUC for all dependent variables. A two-way repeated measures ANOVA (trial x time) was also used to determine differences for glucose, insulin, glucagon, GIP, GLP-1, and C-peptide between timepoints for each trial. Greenhouse Geisser correction was used if the assumption of sphericity was violated and a Bonferroni post-hoc test was used for making comparisons when appropriate. The level of significance was set at *p* ≤ 0.05. All statistical analyses were performed using SPSS statistical software (IBM™ SPSS Statistics v.25, Armonk, NY, USA). All data are expressed as mean ± standard error (SE).

## Results

Fasting values for C-peptide were higher for W (1217.7 ± 21.8 pg/mL) compared to EX (1034.2 ± 165.4 pg/mL; *p* = 0.02). There were no other differences for fasting variables across the four trials (Table 3).

**Table 3:** Fasting values for variables between all four trials

	R	EX	W	EXW
Glucose (mmol/L)	4.97 ± 0.1	4.84 ± 0.1	5.01 ± 0.2	4.93 ± 0.1
Insulin (pg/mL)	945.6 ± 153.2	833.5 ± 149.1	895.9 ± 163.9	917.9 ± 148.7
C-peptide (pg/mL)	1235.5 ± 230.5	1034.2 ± 165.4	1217.7 ± 201.8^b^	1173.7 ± 191.0
GIP (pg/mL)	73.6 ± 9.7	75.3 ± 11.1	66.8 ± 7.8	81.9 ± 14.7
GLP-1 (pg/mL)	7.8 ± 2.0	6.7 ± 0.9	6.5 ± 0.8	7.9 ± 2.7
Glucagon (pg/mL)	78.1 ± 11.1	73.2 ± 9.2	66.6 ± 7.5	76.7 ± 10.7

^b^Represents a significant difference compared to EX. Data are represented as mean ± SE

### Glucose

**Fig. 3:**
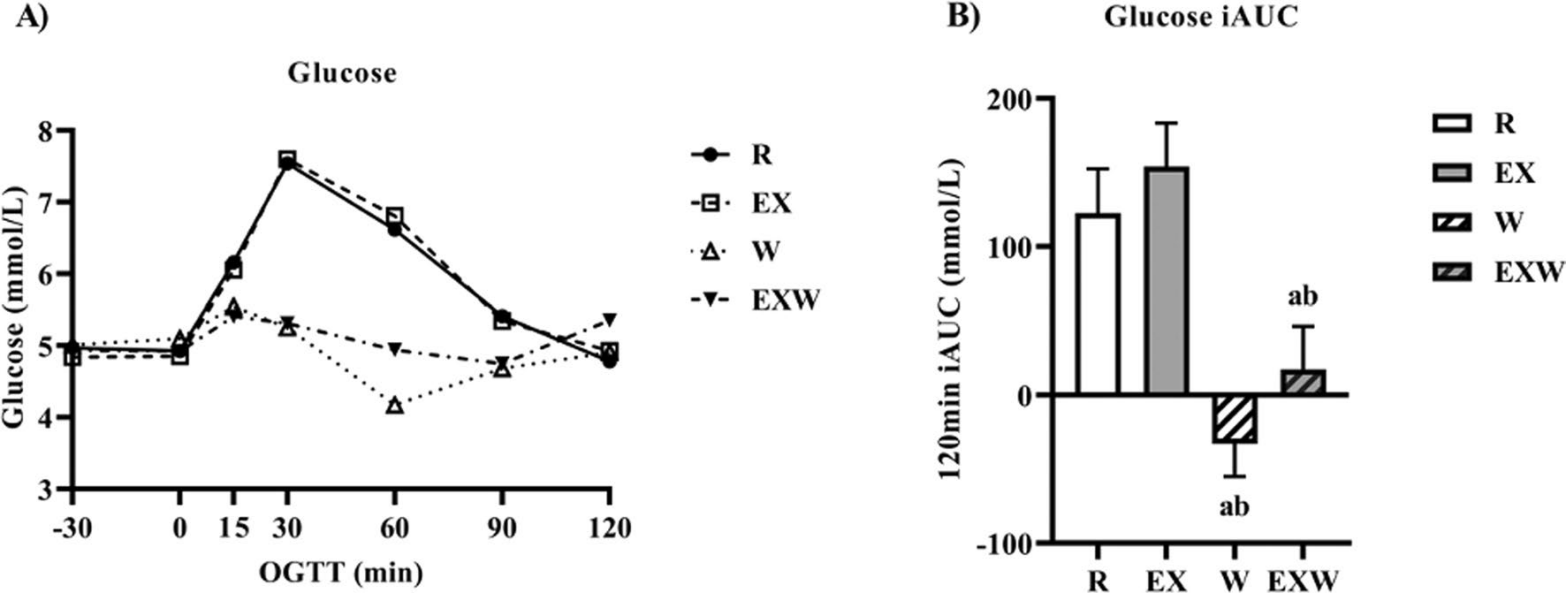
**A** Blood glucose response (mmol/L) prior to and during a two-hour oral glucose tolerance test between each trial. **B** Blood glucose iAUC (mmol/L) during the two-hour oral glucose tolerance test between each trial. *R*; no exercise, no whey protein, EX; exercise, no whey protein, *W*; no exercise, whey protein, EXW; exercise, whey protein. ^a^Represents a significant difference compared to *R*. ^b^Represents a significant difference compared to EX

No differences in glucose values were reported between timepoints for each trial (Fig. 3A). There was a main effect observed for glucose iAUC. Glucose iAUC was lower for W (− 32.9 ± 22.3 mmol/L) compared to R (122.7 ± 29.8 mmol/L; *p* < 0.01) and EX (154.3 ± 29.2 mmol/L;

*p* < 0.01; Fig. 3B). Additionally, glucose iAUC was lower for EXW (17.4 ± 28.9 mmol/L) compared to R (*p* < 0.01) and EX (*p* < 0.01). There were no differences between W and EXW.

### Insulin and C-peptide

**Fig. 4:**
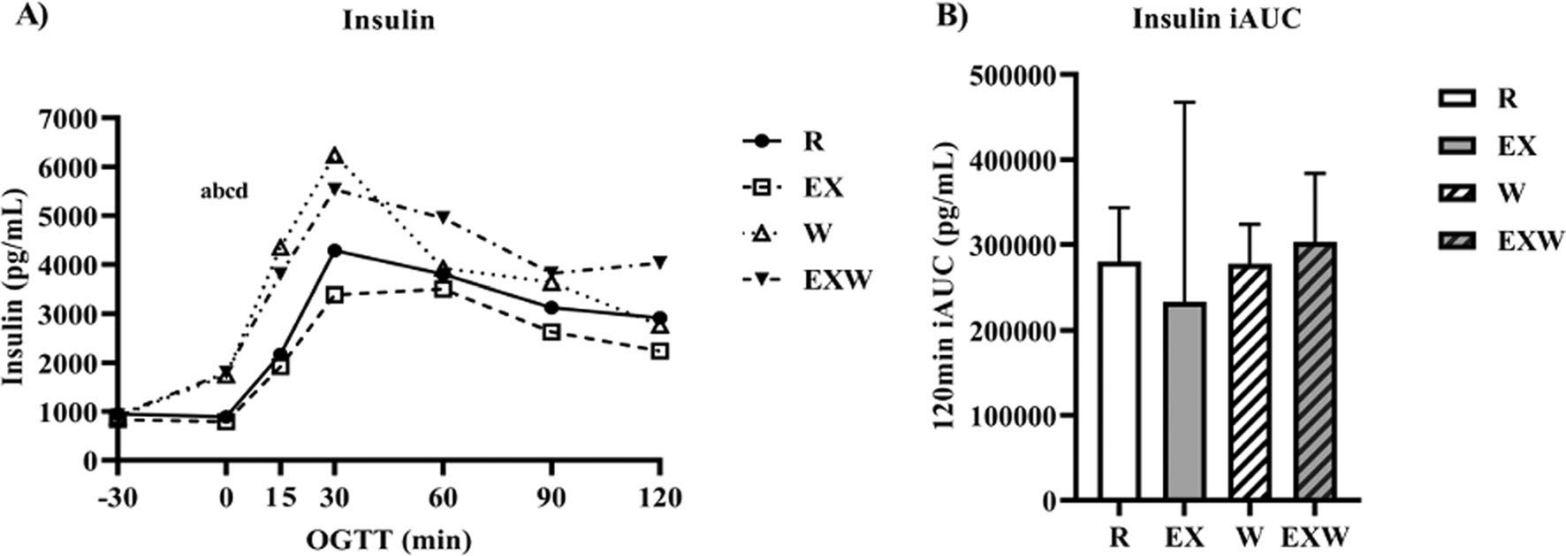
**A** Insulin response (pg/mL) prior to and during a two-hour oral glucose tolerance test between each trial. **B** Insulin iAUC (pg/mL) during the two-hour oral glucose tolerance test between each trial. *R*; no exercise, no whey protein, EX; exercise, no whey protein, *W*; no exercise, whey protein, EXW; exercise, whey protein. ^a^ and ^b^ represent significant differences for W compared to *R* and EX between timepoints -30 to 0, respectively. ^c^ and ^d^ represent significant differences for EXW compared to R and EX between timepoints −30 to 0, respectively

**Fig. 5:**
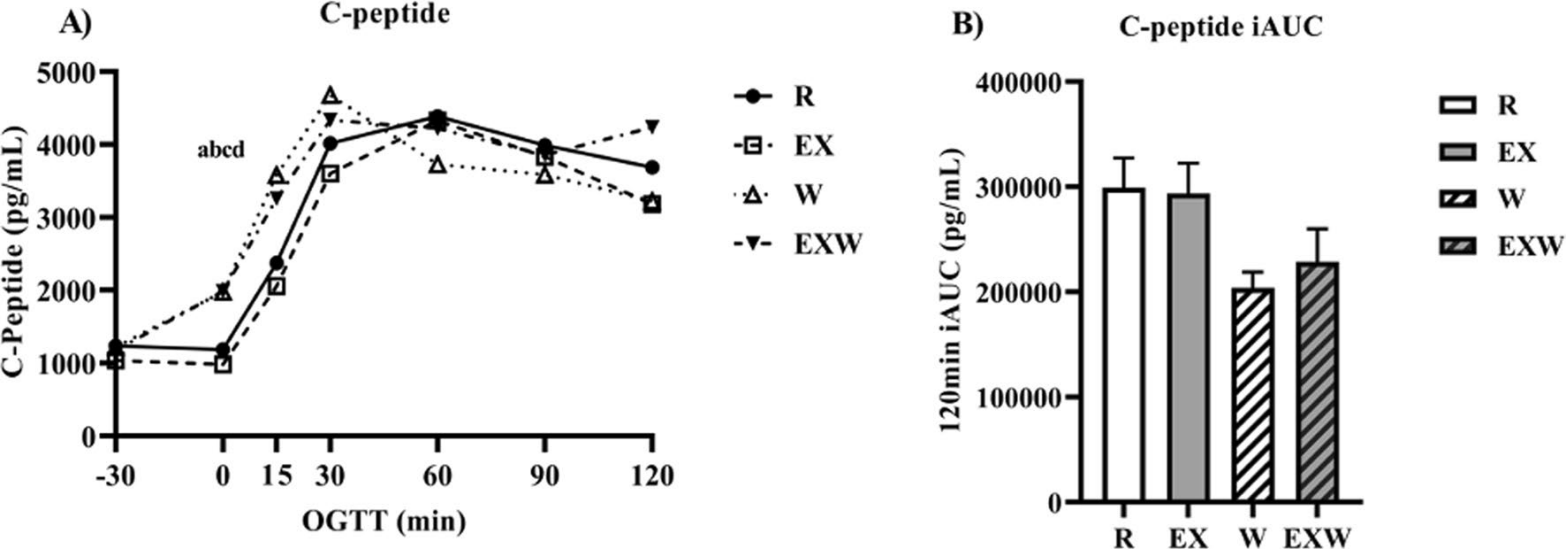
**A** C-peptide response (pg/mL) prior to and during a two-hour oral glucose tolerance test between each trial. **B** C-peptide iAUC (pg/mL) during the two-hour oral glucose tolerance test between each trial. *R*; no exercise, no whey protein, EX; exercise, no whey protein, *W*; no exercise, whey protein, EXW; exercise, whey protein. ^a^ and ^b^ represent significant differences for *W* compared to *R* and EX between timepoints -30 to 0, respectively. ^c^ and ^d^ represent significant differences for EXW compared to R and EX between timepoints compared to R and EX between −30 to 0, respectively

As shown in Fig. 4A, insulin was elevated during the 30-min whey protein preload period (timepoints -30 to 0) for both W (1742.0 ± 238.0 pg/mL) and EXW (1791.4 ± 292.8 pg/mL) compared to R (891.6 ± 165.2 pg/mL; *p* < 0.01) and EX (789.1 ± 147.2 pg/mL; *p* < 0.01). After consuming the 75 g glucose tolerance test drink (timepoint 0 from Fig. 2), there were no differences in insulin iAUC between the four trials. C-peptide response was similar to insulin, with a significant increase between timepoints -30 to 0 for W (1979.0 ± 260.1 pg/mL; *p* = 0.02) and EXW (1984.4 ± 273.6 pg/mL; p < 0.01) compared to EX (982.8 ± 160.6 pg/mL) and R (1183.7 ± 215.6 pg/mL; *p* < 0.01; Fig. 5A). No differences were observed for C-peptide iAUC between the four trials (Fig. 5B).

### Incretins and glucagon

**Fig. 6:**
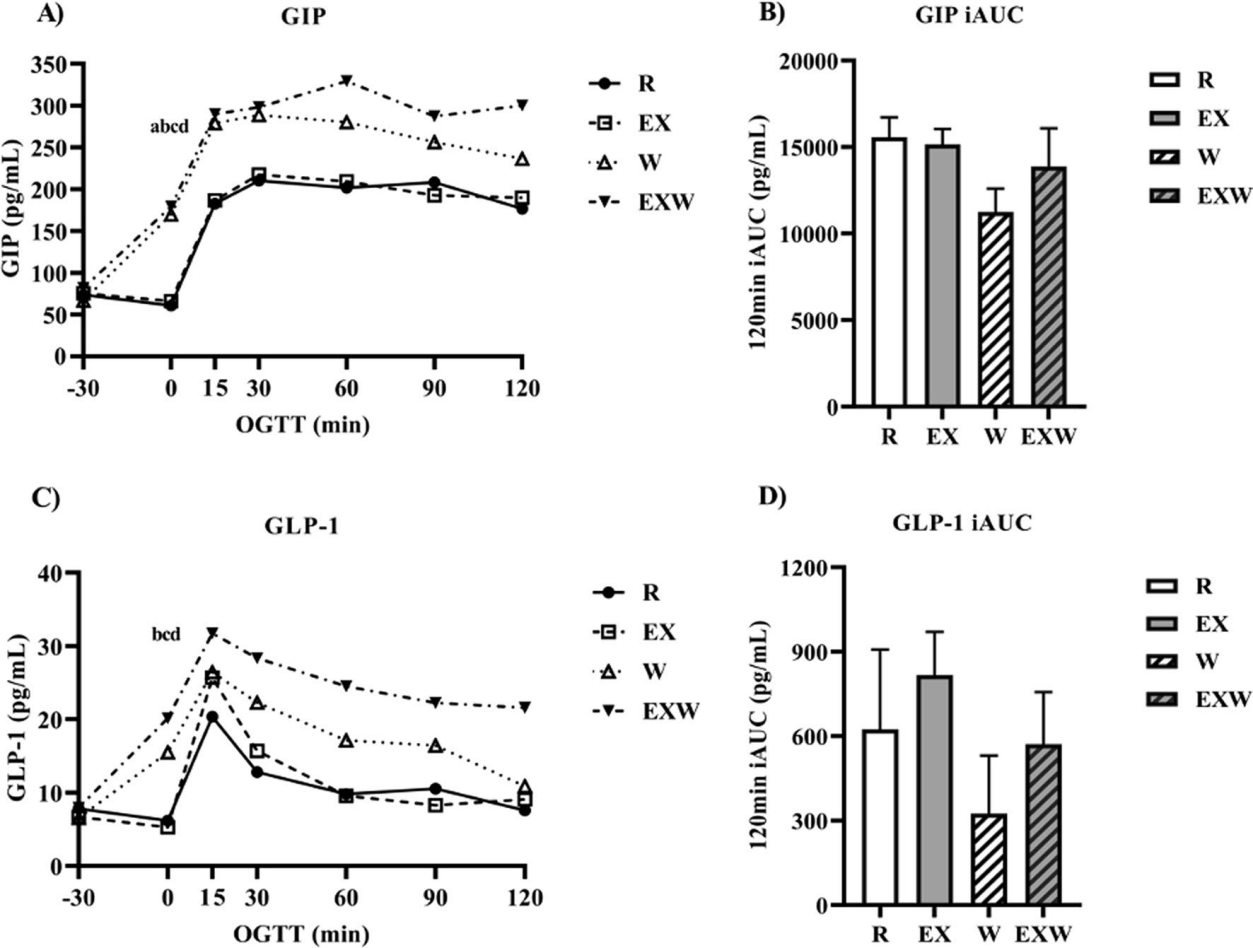
**A** GIP response (pg/mL) prior to and during a two-hour oral glucose tolerance test between each trial. **B** GIP iAUC (pg/mL) during the two-hour oral glucose tolerance test between each trial. **C** GLP-1 response (pg/mL) prior to and during a two-hour oral glucose tolerance test between each trial. **D** GLP-1 iAUC (pg/mL) during the two-hour oral glucose tolerance test between each trial. *R*; no exercise, no whey protein, EX; exercise, no whey protein, *W*; no exercise, whey protein, EXW; exercise, whey protein. ^a^ and ^b^ represent significant differences for W compared to R and EX between timepoints -30 to 0, respectively. ^c^ and ^d^ represent significant differences for EXW compared to R and EX between timepoints −30 to 0, respectively

As shown in Fig. 6A, GIP was elevated during the 30-min whey protein preload period (timepoints -30 to 0) for W (169.5 ± 19.5 pg/mL) and EXW (179.3 ± 11.7 pg/mL) compared to R (60.9 ± 8.1 pg/mL; *p* < 0.01) and EX (66.1 ± 7.6 pg/mL; *p* < 0.01). Similarly, GLP-1 was elevated during the 30-min whey protein period (timepoints -30 to 0) for W (15.5 ± 2.5 pg/mL) compared to EX (5.3 ± 1.4 pg/mLl *p* < 0.01), while EXW was increased during this period compared to R (6.2 ± 1.8 pg/mL; *p* < 0.01) and EX (*p* < 0.01; Fig. 6C.) There were no significant differences in iAUC between the four trials for GIP (Fig. 6B) or GLP-1 (Fig. 6D).

Glucagon was elevated during the 30-min whey protein preload period (timepoints -30 to 0) for both W (140.9 ± 11.6 pg/mL) and EXW (163.5 ± 14.9 pg/mL) compared to R (67.7 ± 

**Fig. 7:**
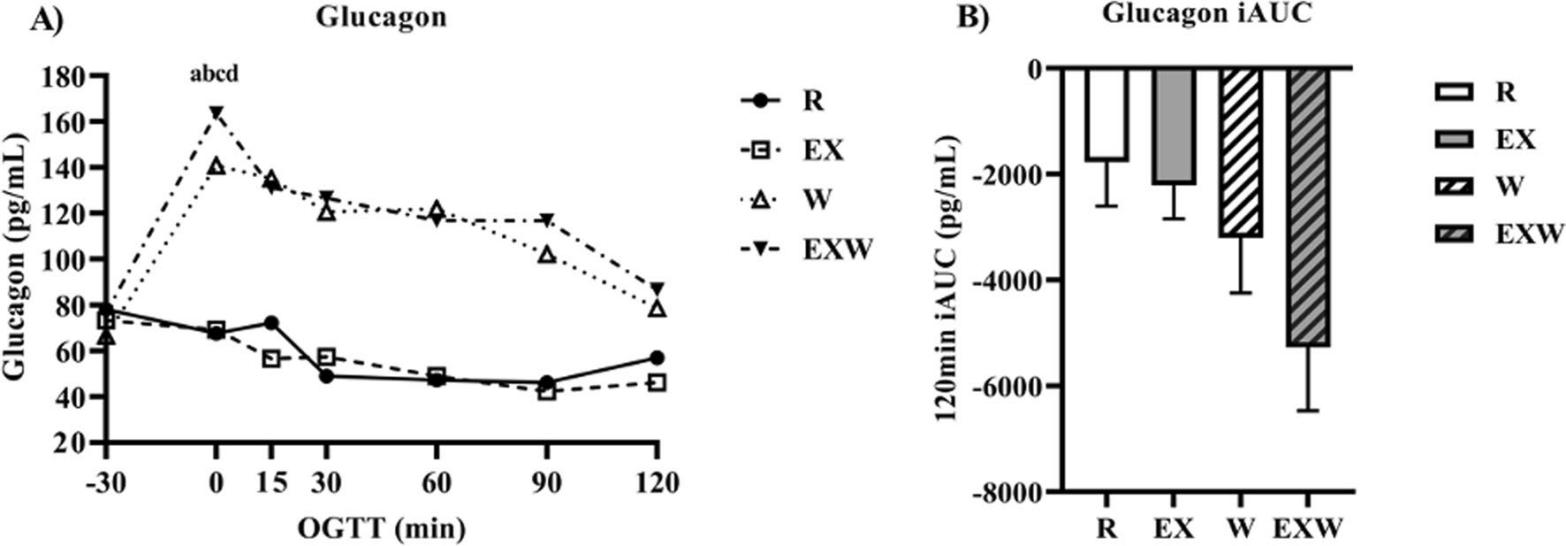
**A** Glucagon response (pg/mL) prior to and during a two-hour oral glucose tolerance test between each trial. **B** Glucagon iAUC (pg/mL) during the two-hour oral glucose tolerance test between each trial. *R*; no exercise, no whey protein, EX; exercise, no whey protein, *W*; no exercise, whey protein, EXW; exercise, whey protein. ^a^ and ^b^ represent significant differences for W compared to R and EX between timepoints -30 to 0, respectively. ^c^ and ^d^ represent significant differences for EXW compared to R and EX between timepoints -30 to 0, respectively

10.3; *p* < 0.01) and EX (69.2 ± 11.2 pg/mL; *p* < 0.01; Fig. 7A). There were no significant differences in iAUC between the four trials for glucagon (Fig. 7B).

## Discussion

Here, we investigated how a single bout of vigorous-intensity aerobic exercise and whey protein consumption, independent of each other and combined, influence glycemic responses to a 75 g OGTT in young adult men. This study is novel as it is one of few studies that has investigated their combined effects on acute glycemia. Moreover, this study utilized a unique time frame (exercise and whey protein separated by 12–14 h) for investigating how aerobic exercise and whey protein interact to affect glycemia.

We found the combination of vigorous-intensity aerobic exercise and whey protein (EXW) reduced glucose iAUC values during a two-hour OGTT compared to exercise alone (EX). Additionally, we observed a similar effect on glucose iAUC for W compared to EX. Importantly, these effects on glucose iAUC were achieved without significant increases in insulin iAUC during W or EXW compared to R or EX. Despite these effects on glucose and insulin iAUC, there were no differences in C-peptide, glucagon, GIP, or GLP-1 iAUC values throughout the two-hour OGTT between the four trials.

### Glucose/insulin

Our results for glucose iAUC when 50 g of whey protein was consumed as a preload (W and EXW) are similar to other studies that have investigated whey protein’s effects at higher doses (50 g) on glycemia [27, 28]. However, these effects on glycemia were not observed when exercise was performed independent of whey protein (EX). Comparably, a previous study reported similar outcomes for glucose and insulin with 31 g of whey protein was consumed as a preload. The authors reported glucose response was reduced during a 75 g OGTT when the whey protein was consumed 15 min prior to a graded exercise test in healthy young men and women, when compared to control and fructose beverages [32]. The improvements in glucose iAUC for W and EXW were accompanied by increases in insulin responses 30 min after whey protein consumption (-30 to 0 timepoint), though insulin iAUC was not different between the four trials. The changes observed for W and EXW may be attributed to this acute spike in insulin following the whey protein preload, which was then followed by a more normalized insulin response throughout the remaining duration of the OGTT.

Whey protein’s positive effects on glucose management are exemplified by our results for EX. EX had similar glucose responses to R at each individual timepoint throughout the OGTT (Fig. 3A), and glucose iAUC values for EX were higher than R (Fig. 3B). Our results for EX are similar to previous studies that have investigated the influence of acute aerobic exercise on glycemia [33, 34]. Though overall insulin responses to the OGTT were lowest for EX, there were no significant effects on insulin iAUC or differences between timepoints compared to the other three trials. We found this surprising, as several studies have reported improvements in insulin responses to aerobic exercise [35, 36]. Our results for EX may be explained most clearly by the fact that the exercise was performed 12–14 h prior to the OGTT, potentially diminishing exercise’s insulin sensitizing effects. As the participants in this study were relatively young, healthy men, it indicates higher intensities and/or durations may be necessary to sustain post-exercise-induced improvements in insulin sensitivity over longer durations (12 + hours) in this population. Moreover, there was large variability in insulin iAUC for EX, which may be explained by elevated insulin concentrations in two participants at several consecutive timepoints during the OGTT for this specific trial. This variability, in addition to a smaller sample size, likely influenced any observed significance of EX on insulin iAUC. Overall, our findings for W in relation to EX indicate whey protein has important applications for managing glycemia. As there were no further improvements with EXW compared to W, our results suggest whey protein may have a larger influence on acute glycemia compared to exercise in this population.

### GIP, GLP-1, and glucagon

The incretins, GIP and GLP-1, have important roles in promoting insulin secretion and glucose management, with some suggesting the incretins account for 50–70% of insulin secretion following a meal [37]. In comparison to previous literature on this topic, Ma et al. (2009) reported whey protein had stimulatory effects on both GIP and GLP-1 at doses similar to those used in this study [27]. We found no differences in iAUC for GIP or GLP-1 between the four trials. However, we observed significant increases in GIP and GLP-1 for both W and EXW during the whey protein preload prior to the OGTT (timepoints -30 to 0). These increases in GIP and GLP-1 for W and EXW coincide with observed changes in insulin, which likely contributed to the improvements in glucose iAUC for these trials. Specifically, we speculate that this brief increase in GIP and GLP-1 following whey protein consumption could have potentiated insulin secretion, contributing to the observed glucose responses for W and EXW. Our findings for GIP and GLP-1 following whey protein consumption is similar to results from Jakubowicz et al. (2014). Using a similar study design, they found increases in GLP-1 30 min after consuming whey protein, though also reporting GLP-1 continued to increase following a high glycemic-index meal [15].

In addition to their effects on insulin secretion, the incretin hormones have regulatory effects on glucagon. Specifically, GIP can enhance glucagon secretion, while GLP-1 has been shown to inhibit glucagon secretion [25, 38]. We found no significant differences in glucagon iAUC between the four trials. However, similar to GIP and GLP-1, when examining glucagon’s response prior to the OGTT (timepoints -30 to 0), glucagon was elevated for both W and EXW during this time period. Considering GIP and GLP-1’s opposing effects on glucagon, it appears GIP may have a stronger regulatory effect on glucagon than GLP-1, contributing to the observed increase in glucagon following the whey protein preload. Comparing responses for glucose, insulin, and glucagon throughout the OGTT, an interesting pattern emerges whereby insulin and glucagon are elevated similarly for W and EXW with respect to R and EX. Specifically, for W and EXW, glucagon peaked after the whey protein preload period (timepoints -30 to 0) but remained elevated, albeit to a lesser extent, throughout the duration of the OGTT. Insulin response was similar, increasing after the whey protein was consumed, though its concentration peaked after the OGTT commenced (30-min timepoint). What is interesting is that despite these similar, yet opposing, responses between glucagon and insulin, glucose responses for W and EXW were relatively stagnant, and even decreased below baseline glucose values throughout the OGTT. This observed effect appears to be exclusive to whey protein consumption as well, as glucagon decreased for EX during the OGTT. What this dynamic interaction between glucagon and insulin means in the context of glycemia regulation when whey protein is consumed, as well as how GIP and GLP-1 may influence these observed effects requires further exploration.

## Limitations

This study and its results have some limitations that should be considered for prospective investigations. For instance, the participants in this study were limited to young, adult men. As the scope of this study is largely relevant to the management of metabolic disease (i.e., type II diabetes), future studies should consider exploring the relationship between whey protein and exercise in individuals with metabolic disease. Additionally, several studies have explored whey protein’s effects on glycemia at varied doses (e.g., 20 g or 30 g). Based upon our findings in this study at 50 g of whey protein, it may be worth exploring how varied doses of whey protein, in conjunction with vigorous-intensity aerobic exercise, may influence glycemia. Lastly, as participants in this study walked at a vigorous intensity for the exercise intervention, our results are limited to this specific type of exercise. It may be worth investigating how different modes of exercise incorporating mechanical loading (e.g., resistance exercise, or aerobic and resistance exercise combined) when combined with whey protein may influence glycemia.

## Conclusions

We found whey protein consumed independent of performing exercise improved glucose responses to a 75 g OGTT. This improvement was also observed when whey protein and vigorous-intensity aerobic exercise were combined, but this effect was lost when exercise was performed alone. These results provide information for how vigorous-intensity aerobic exercise and whey protein individually, and when combined, may affect acute glycemia. This study addressed how the timing between exercise and whey protein may influence these responses as well, which is important when considering the real-world application of these results. To summarize, our results highlight the short-term effectiveness of whey protein, independently and in combination with aerobic exercise, for improving glycemia.

## Data Availability

The datasets used and/or analyzed in this current study are available from the corresponding author on reasonable request.

## Abbreviations

DEXA: Dual energy X-ray absorptiometry
EX: Exercise/no whey protein
EXW: Exercise/whey protein
GIP: Gastric inhibitory peptide
GLP-1: Glucagon-like peptide 1
_i_AUC: Incremental area under the curve
OGTT: Oral glucose tolerance test
R: No exercise/no whey protein
W: No exercise, whey protein
